# Association between Polymorphism eNOS4, tPA, Factor V Leiden, Prothrombin, and Methylenetetrahydrofolate Reductase and the Occurrence of Legg–Calvé–Perthes Disease

**DOI:** 10.3390/jcm12165209

**Published:** 2023-08-10

**Authors:** Anna Matuszewska, Oliwer Sygacz, Łukasz Matuszewski, Szymon Stec, Andrzej Grzegorzewski, Jacek Gągała

**Affiliations:** 1 Department of Biochemistry, Maria Curie-Skłodowska University, 20-033 Lublin, Poland; 2 Department of Paediatric Orthopaedics and Rehabilitation, Medical University of Lublin, 20-059 Lublin, Poland; oliwer.sygacz@gmail.com (O.S.); lukasz.matuszewski@umlub.pl (Ł.M.); szmnstec@gmail.com (S.S.); 3 Clinic of Orthopaedics and Paediatric Orthopaedic, Medical University of Łódź, 90-419 Lodz, Poland; 4 Orthopedic Surgery and Traumatology Department, Medical University of Lublin, 20-059 Lublin, Poland; jacekgagala@gmail.com

**Keywords:** Legg–Calvé–Perthes disease, pathogenesis, genetics, risk factors

## Abstract

Background. Legg–Calvé–Perthes (LCPD) disease is a complex condition affecting the femoral head’s epiphysis in children. It occurs with a prevalence ranging from 0.4 to 29.0 cases per 100,000 children under the age of 15. It involves various factors, including genes associated with coagulation and fibrinolysis, pro-inflammatory factors, and vasoactive substances. Methods. We investigated the relationship between genetic mutations associated with coagulation and vascular disorders and the occurrence of LCPD in Polish patients. We performed a study involving 25 patients with LCPD and 100 healthy controls. All subjects were genotyped for eNOS4, Factor V Leiden, prothrombin, tPA25, and MTHFR polymorphism. Results. The analysis revealed that the frequencies of eNOS4 genotypes were significantly different in LCPD patients than in the control group (*p* = 0.018). The frequencies of 4a allele were significantly higher in patients with LCPD than in the healthy population (26% vs. 9%, *p* = 0.0012). There were no significant differences in genotype and allele frequencies for Factor V Leiden, prothrombin tPA 25, and MTHFR gene polymorphisms between patients with LCPD and the controls. Conclusions. Genotype and allele frequencies of eNOS4 were significantly higher in patients with LCPD. These findings suggest a potential association between the eNOS gene polymorphism and an increased risk of developing LCPD.

## 1. Introduction

Legg–Calvé–Perthes disease (LCPD) is a complex disease involving the epiphysis of the femoral head in the pediatric population. Historically, it was considered an osteochondrosis; however, today it is referred to as idiopathic aseptic necrosis of the femoral head in children. The prevalence of LCPD is estimated at 0.4/100,000 to 29.0/100,000 children under 15 years of age, with the peak incidence in children aged 4 to 8 years [1]. If left untreated, developing degenerative changes associated with arthritis may eventually lead to total hip arthroplasty. Therefore, early diagnosis and treatment are crucial to prevent the collapse of the femoral head, progressive femoral head deformity, and functional impact [2].

Although the etiology of LCPD has been extensively studied, it remains poorly understood. Among the main hypotheses for the epidemiology of this disease, a multifactorial genesis is proposed, involving the contribution of genetic, mechanical, and systemic conditions to the pathogenesis of femoral head osteonecrosis. The best-supported theory suggests that the proper blood supply to the epiphysis is disturbed due to repeated mechanical stresses [3,4]. Episodes of interrupted blood supply to the femoral head may happen repeatedly, either as single events or multiple occurrences. When the blood flow to the femoral head is compromised, it sets off a sequence of events within and around the femoral head. This results in avascular necrosis, which can affect a part or the entire epiphysis. Osteoclasts absorb the necrotic bone, and to repair the damage, woven bone is formed around the edges of the epiphysis. Gradually, this woven bone is replaced by mature lamellar bone, leading to complete healing of the epiphysis. At the same time, changes occur outside the femoral head, including hypertrophy of the synovium, ligamentum teres, and articular cartilage. These soft tissue alterations, combined with muscle spasms, trigger the extrusion of the femoral head. This extrusion exposes the femoral head to stresses passing through the acetabular margin, ultimately resulting in irreversible deformation of the femoral head. Slight modifications to the femoral head might take place. These changes can result in lasting deformities of the femoral head and a lack of proper alignment in the joint, which will remain present throughout an individual’s lifetime [2].

Additionally, several environmental factors have been implicated, with smoking being the most frequently reported risk factor for the development of LCPD [5,6,7,8]. Interestingly, smoking by the mother has also been shown to affect the baby’s risk of developing the disease. Moreover, evidence has been presented of an association between environmental smoke and LCPD, both during maternal pregnancy and in the patient’s childhood. Low birth weight has been identified as a potential risk factor for Perthes disease (LCPD) in some research studies [9]. Other potential predisposing factors are attention deficit hyperactivity disorder (ADHD) and mental stress, which may also play a role in the development of LCPD [10]. Furthermore, obesity has been positively associated with LCPD, with a correlation observed between higher obesity levels and more severe clinical signs and deformity of the femoral head [11]. A study by Lee et al. [12] reported significantly higher leptin levels in LCPD patients, and the levels correlated with the disease’s stage.

Scientific reports also suggest a genetic role in the development of LCPD. Many gene polymorphic variants may increase the risk of LCPD development in children due to their influence on atherothrombosis and fibrinolysis. Notably, Miyamoto et al. [13] were the first to describe a case of LCPD associated with a mutation of the collagen type II gene (COL2A1). Additionally, Li et al. [14] conducted a study involving a four-generation family and identified mutations in six family members affected with the disease.

In the pathogenesis of LCPD, susceptibility genes are most often investigated among those related to the coagulation and fibrinolysis system, renin–angiotensin–aldosterone system, pro-inflammatory factors, and vasoactive substances. The literature reports associations of LCPD with gene mutations such as Factor-V Leiden, prothrombin G20210A, methylenetetrahydrofolate reductase (MTHFR) C677T, endothelial nitric oxide synthase (eNOS), and plasminogen activator inhibitor-1 4G/5G [15,16,17].

Factor V Leiden is a genetic mutation in the F5 gene that results in an altered form of factor V, a protein involved in promoting prothrombin activation and thrombin generation, critical processes in blood clotting. This mutation leads to Factor V Leiden thrombophilia, making it the most prevalent inherited cause of thrombophilia, characterized by an increased risk of abnormal blood clot formation [15].

Prothrombin is a vitamin K-dependent glycoprotein synthesized in the liver. It serves as a precursor to the serine protease thrombin and plays a crucial role in regulating hemostasis and thrombosis processes. The G20210A mutation in the prothrombin gene is a common genetic variant associated with increased thrombotic risk. This single-nucleotide polymorphism (SNP) occurs in a noncoding region of the prothrombin gene, where guanine (G) is replaced by an adenine (A) at nucleotide position 20210 in the 3′-untranslated region [18].

Methylenetetrahydrofolate reductase (MTHFR) is a vital enzyme involved in homocysteine metabolism. Its primary role is to convert homocysteine to methionine through remethylation. Genetic mutations in the MTHFR gene can lead to reduced enzymatic activity, resulting in increased levels of homocysteine (hyperhomocysteinemia) and potential vascular complications. One common mutation known as C677T in the MTHFR gene leads to a substitution of cytidine with thymidine at nucleotide position 677 [19].

Tissue-type plasminogen activator (tPA) is a crucial component of the fibrinolytic system responsible for converting plasminogen to plasmin when fibrin is present. Plasmin, in turn, plays a vital role in breaking down the fibrin clot, leading to the formation of soluble fibrin degradation products. The possible association between the tPA Alu I/D genotype and arterial or venous thromboembolism has been investigated in various clinical studies, yielding mixed results [20].

Nitric oxide (NO) is a vital intracellular messenger involved in vascular function and bone turnover. Endothelial nitric oxide synthase (eNOS) is a key enzyme responsible for NO production. Polymorphisms in the eNOS gene, including the eNOS4a allele, have been linked to lower NO levels. Reduced NO production can contribute to problems of angiogenesis, thrombosis, and bone turnover [21].

Given the complexity of LCPD and its potential genetic basis, this study aims to assess the correlation between genetic mutations associated with coagulation and vascular disorders. Therefore, we suggest that polymorphism of eNOS4, tPA, Factor V Leiden, prothrombin, and methylenetetrahydrofolate reductase may lead to the development of Legg–Calvé–Perthes disease (LCPD) in a population of Polish patients.

## 2. Materials and Methods

### 2.1. Patients

A cohort of 25 unrelated young patients aged 4–12 participated in this study. All patients were of Caucasian ethnicity and of Polish descent, originating from the eastern region of Poland. They were admitted to the Orthopedics and Rehabilitation Department for the diagnosis and treatment of Legg–Calvé–Perthes disease (LCPD). The diagnosis of LCPD was established through clinical examination and radiographic analysis. The Herring classification [22] was utilized for radiographic assessment, with anteroposterior (AP) and axial view X-rays taken for both hips of all patients. LCPD was observed in one hip of each patient. Based on the Herring classification, group A was identified in six hips, group B in fifteen hips, and group C in four hips.

A control group of healthy adult individuals (*n* = 100) without any indications, previous history, or familial history of LCPD was recruited from among blood donors and hospital personnel. Like the patient group, all participants in the control group also originated from the eastern part of Poland. The control group consisted of 20 women and 80 men, with an average age of 36.6 years (ranging from 20 to 55 years).

A written informed consent for genetic studies was obtained from all the patients and subjects from the control group. The study protocol was evaluated and approved by the Ethics Committee at the Medical University in Lublin (KE-0254/67/2018).

### 2.2. Genetic Testing

**Figure 1:**
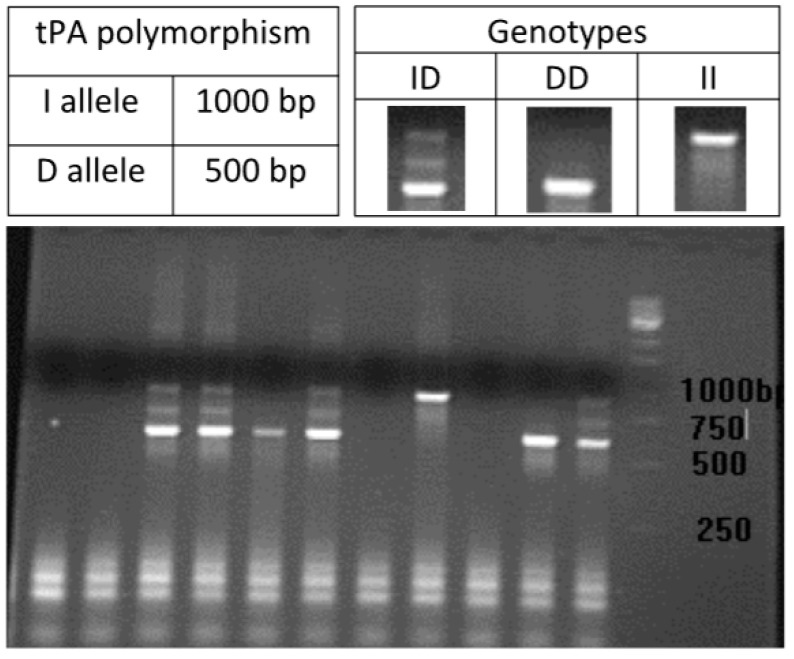
PCR product of the polymorphism in PLAT tPA 25 gene.

**Figure 2:**
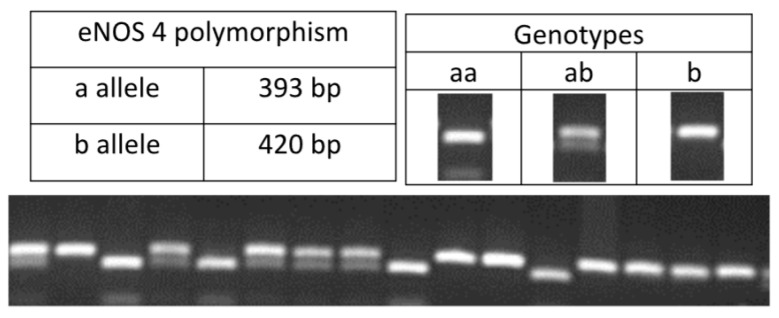
PCR product of the polymorphism in intron 4 of the eNOS gene.

**Figure 3:**
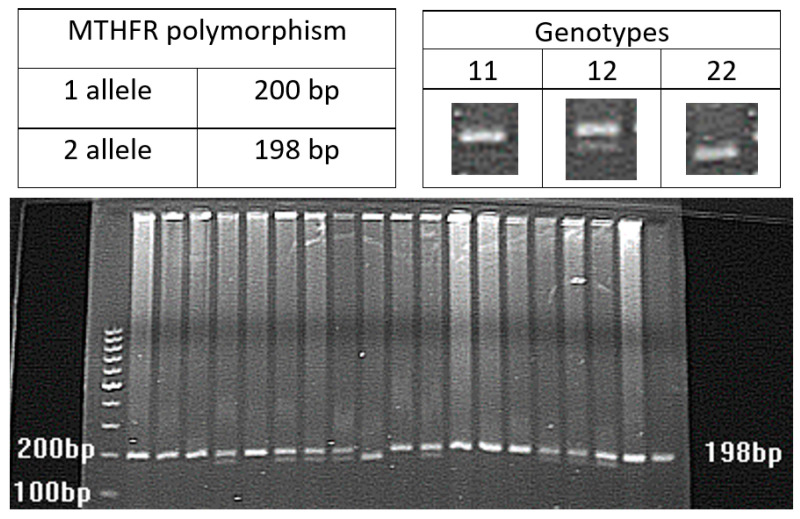
PCR product of the polymorphism in C667T of the MTHFR gene.

Genomic DNA was extracted from peripheral blood leukocytes using the protocol outlined by Madisen et al. [23], with slight adjustments. The genotyping of samples was performed through polymerase chain reaction (PCR) using the methods previously documented by the authors [18,19,21,24,25], as listed in Table 1. The PCR products were analyzed by electrophoresis (Figure 1, Figure 2 and Figure 3).

### 2.3. Statistical Analysis

All calculations were conducted using SPSS for Windows 17.0. Normally distributed data are reported as means ± standard deviation (SD). To compare genotype distribution and allele frequencies between groups, the chi-square test and Fisher’s exact test were employed, respectively. Odds ratios (ORs) and 95% confidence intervals (CIs) were calculated using appropriate 2 × 2 contingency tables.

## 3. Results

We genotyped the polymorphism of five genes associated with excessive clot formation, a disorder of clot dissolution, and a gene responsible for nitric oxide synthesis (Factor V Leiden, Prothrombin, MTHFR, tPA 25, eNOS) in 25 patients with LCPD and 100 healthy controls. The mean age of patients was 8.6 ± 2.46 years. There were 23 boys (92%) and 2 girls (8%) in the study group. All patients had one affected hip. Genotype and allele frequencies of LCPD patients are shown in Table 2 and Table 3.

The analysis revealed significant differences in the frequencies of the eNOS4 genotype between patients with Legg–Calvé–Perthes disease (LCPD) and the control group. When compared to the control group, the frequency of the 4a allele was found to be higher in patients with LCPD (26% vs. 9%, *p* = 0.0012, odds ratio [OR] = 3.55, 95% CI 1.60–7.87). However, there were no significant differences in allele frequencies for Factor V Leiden, prothrombin tPA 25, and MTHFR gene polymorphisms between patients with LCPD and the control group.

Regarding genotypes, the frequency of the 4a/a genotype in patients with LCPD was higher than in the controls, although the difference was not statistically significant (8% vs. 1%, *p* = 0.05146). However, the frequency of the 4a/b genotype was found to be statistically significant (*p* = 0.018, OR = 3.33, 95% CI 1.23–9.00) among patients with LCPD compared to the controls. Like the allele frequencies, there were no significant differences in genotype frequencies for Factor V Leiden, prothrombin, tPA 25, and MTHFR gene polymorphisms between patients with LCPD and the controls.

## 4. Discussion

Legg–Calvé–Perthes disease (LCPD) is a complex condition with the exact underlying causes still not fully understood. However, it is believed that recurrent disruptions in the blood supply to the femoral head play a crucial role in its pathogenesis, leading to ischemic necrosis, characterized by insufficient blood flow and tissue death, resulting in pathological changes and structural abnormalities in the femoral head [26,27].

In our study, we observed that the genotype and allele frequencies of eNOS4 were notably higher in patients with LCPD. These results indicate a possible link between the eNOS4 gene polymorphism and elevated susceptibility to developing LCPD. Conversely, we did not find any substantial differences in genotype and allele frequencies for Factor V Leiden, prothrombin tPA 25, and MTHFR gene polymorphisms between patients with LCPD and the control group.

One important factor involved in regulating internal hemodynamics and vascular function is nitric oxide (NO), synthesized by endothelial nitric oxide synthase (eNOS). Genetic polymorphisms within the eNOS gene have been investigated for their potential impact on eNOS expression and NO production [24]. Specifically, three polymorphisms have been identified, including −786T > C (rs2070744) in the promoter region, 894G > T (rs1799983) in exon 7, and a variable number of tandem 4a4b repeats (rs61722009) in intron 4 [28].

Recently, Zhao et al. conducted a study investigating the potential link between eNOS gene polymorphisms and LCPD occurrence. Specifically, they investigated the association of a 27-bp variable number tandem repeat (VNTR) polymorphism in intron 4 and a G894T polymorphism in exon 7 of the endothelial nitric oxide synthase (eNOS) gene with LCPD. Regarding the 27-bp VNTR polymorphism, individuals diagnosed with LCPD exhibited a higher prevalence of the ab genotype compared to the healthy control group. Additionally, regarding the G894T polymorphism, the LCPD case group showed a higher frequency of the heterozygous genotype GT compared to the control group consisting of healthy individuals. The results indicate that these eNOS gene polymorphisms may be a risk factor for LCPD. [17] Similarly, an Iranian study by Azarpira et al. revealed an association between the eNOS 894G > T and −786T > C polymorphisms and LCPD in Iranian children [29].

Prothrombin is a precursor to thrombin, an enzyme essential for blood clotting. The G20210A prothrombin gene mutation leads to higher prothrombin levels in the bloodstream, resulting in increased thrombin production and thrombophilia [30]. Studies have presented conflicting findings regarding the association between the PT G20210A mutation and LCPD. While a Mexican study by Buendia-Pazaran et al. and Hayek et al. found no relationship [31,32], Vosmaer et al. from the Netherlands reported an increased incidence of LCPD in the presence of the prothrombin G20210A mutation [33].

The methylenetetrahydrofolate reductase enzyme (MTHFR) regulates homocysteine (Hcy) levels in the bloodstream. Alterations in the MTHFR gene, particularly the C677T polymorphism, have been associated with changes in Hcy levels, which in turn may increase the risk of prothrombotic states [34]. The association between MTHFR polymorphisms and LCPD has yielded mixed results. While Buendia-Pazaran et al. found an association with the MTHFR rs1801133 polymorphism in Mexican children [31], other studies in Brazilian and Iranian populations did not find a significant link [32,35]. Additionally, García-Alfaro et al. reported that the rs1801133(T) variant of the MTHFR gene was associated with more severe forms of LCPD (Catterall III-IV) [36].

Factor V Leiden is a variant of a protein called factor V that plays a role in promoting prothrombin activation and thrombin generation, important processes in blood clotting. Factor V Leiden mutation in the F5 gene causes Factor V Leiden thrombophilia. Factor V Leiden is the most common inherited cause of thrombophilia, with an average prevalence of the mutation ranging from 4 to 5% in healthy individuals of Caucasian descent [36,37]. Factor V Leiden has been firmly established as a significant and indisputable risk factor for the development of venous thrombosis [24]. Studies by Vosmaer et al. and Szepesi et al. showed an increased incidence of LCPD in the presence of the Factor V Leiden mutation [33,38]. However, other studies, such as those by Worotanarat et al. and Lopez-Franco et al., did not find a significant association [39,40].

Tissue-type plasminogen activator (tPA) is a key component of the fibrinolytic system responsible for breaking down blood clots. It acts by converting plasminogen into the active enzyme plasmin when fibrin is present. Plasmin, in turn, facilitates the digestion of the fibrin clot, resulting in the formation of soluble fibrin degeneration products. Investigation of the tPA gene has identified a prevalent Alu insertion/deletion (I/D) polymorphism located in intron 8 [41]. To our knowledge, the potential association between TPA polymorphisms and Legg–Calvé–Perthes disease (LCPD) has not been previously investigated or studied. We also have not found any association between tPA polymorphism and LCPD.

In conclusion, understanding the genetic and molecular factors involved in LCPD development is complex and often yields varied results. Therefore, exploring the genetic factors associated with this condition is of paramount importance. Understanding the genetic basis of Perthes Disease can provide crucial insights into the disease’s pathogenesis. Genetic testing may enable early detection of individuals at a higher risk of developing LCPD. Studying the genetic basis of Perthes Disease can help assess the risk of the disease within families. Further research and large-scale studies are necessary to shed light on the precise mechanisms and associations underlying this condition.

It is important to acknowledge the limitations of our study, including the small sample size and variations in patient age, which may have influenced the results. Therefore, further investigations employing larger sample sizes and more comprehensive exploration of hypofibrinolysis markers are warranted to corroborate our findings.

## 5. Conclusions

In summary, our study conducted on Polish patients revealed an association between the eNOS 4a gene polymorphism and Legg–Calvé–Perthes disease (LCPD). However, we did not find any evidence supporting the notion that Factor V Leiden, prothrombin G20210A, MTHFR gene, and tPA mutations are risk factors for LCPD in the Polish population.

## Figures and Tables

**Table 1:** Panel of markers included in the study.

Gene	Polymorphism	Type	References
Factor V Leiden	G1691A	RFLP	Zoller et al. [24]
Prothrombin	G20210a	RFLP	Ahmed et al. [21]
MTHFR	C667T	RFLP	Ksiazek et al. [18]
PLAT TPA 25	I/D	Ins.Alu	Toshkoff et al. [19]
eNOS	eNOS intron 4	RFLP	Gagala et al. [25]

**Table 2:** Allele frequencies of five polymorphisms in LCPD patients and controls.

Allele	Controls *N* = 200	Patients *N* = 50
Factor V Leiden		
A	6 (3%)	1 (2%)
G	194 (97%)	49 (98%)
Prothrombin		
A	2 (1%)	1 (2%)
G	198 (99%)	49 (98%)
MTHFR		
2	56 (28%)	13 (26%)
1	144 (72%)	37 (74%)
tPA 25		
D	73 (36.5%)	16 (32%)
I	127 (63.5%)	34 (68%)
eNOS		
4a	18 (9%)	13 (26%) ^1^
4b	182 (91%)	37 (74%)

^1^ *p* = 0.0012, OR = 3.55 95% CI 1.60–7.87.

**Table 3:** Genotype frequencies of five polymorphisms in LCPD patients and controls.

Allele	Controls *N* = 100	Patients *N* = 25
Factor V Leiden		
GA	3 (3%)	1 (4%)
GG	97 (97%)	24 (96%)
Prothrombin		
GA	2 (2%)	1 (4%)
GG	98 (98%)	24 (96%)
MTHFR		
22	7 (7%)	2 (8%)
12	42 (42%)	9 (36%)
11	51 (51%)	14 (56%)
tPA 25		
DD	18 (18%)	4 (16%)
ID	37 (37%)	8 (32%)
II	45 (45%)	13 (52%)
eNOS		
4a/a	1 (1%)	2 (8%)
4a/b	16 (16%)	9 (36%) ^1^
4b/b	83 (83%)	14 (56%)

^1^ *p* = 0.018, OR = 3.33 95%, CI 1.23–9.00.

## Data Availability

Data available in Department of Paediatric Orthopaedics and Rehabilitation, Medical University of Lublin, Lublin, Poland (medical documentation).
